# Telomere length as a predictive marker for long-term cognitive function in a mouse model of subarachnoid hemorrhage

**DOI:** 10.4103/NRR.NRR-D-24-01150

**Published:** 2025-06-19

**Authors:** Qia Zhang, Chaoran Xu, Jiayong Fan, Chengjian Lou, Jiarui Chen, Jianmin Zhang, Jun Mo

**Affiliations:** 1 Department of Neurosurgery, The Fourth Affiliated Hospital of School of Medicine, and International School of Medicine, International Institutes of Medicine, Zhejiang University, Yiwu, Zhejiang Province, China; 2 Department of Neurosurgery, The Second Affiliated Hospital, Zhejiang University School of Medicine, Hangzhou, Zhejiang Province, China; 3 Department of Neurosurgery, First Affiliated Hospital of Wenzhou Medical University, Wenzhou, Zhejiang Province, China

**Keywords:** acetyl-coenzyme A synthetase-2, brain aging, DNA damage response, long-term prognosis, subarachnoid hemorrhage, telomere length

## Abstract

Subarachnoid hemorrhage is a subtype of stroke that causes severe neurological damage and is associated with poor long-term prognosis. Cognitive impairment is a major manifestation of long-term neurological dysfunction in patients with subarachnoid hemorrhage. However, there is notable absence of biological markers to predict long-term prognosis in this patient population. Given the aging-like neurocognitive phenomena associated with subarachnoid hemorrhage, this study postulates that telomere length, a recognized biomarker for aging, could be used as a prognostic indicator for subarachnoid hemorrhage. A left internal carotid artery intravascular puncture mouse model was used to simulate subarachnoid hemorrhage. Comprehensive neurological test scores were obtained through neurobehavioral assessments conducted at one-month intervals. Concurrently, the relative telomere length was analyzed by quantitative polymerase chain reaction, which was performed using DNA extracted from ear notch and brain tissue after each assessment. Furthermore, proteomic analysis was employed to investigate differential protein expression in hippocampal tissue. Subarachnoid hemorrhage mice exhibited persistent neurocognitive impairment over a prolonged period of time. There was a significant positive correlation between telomere length and neurological test scores, confirming the usefulness of telomere length as a prognostic indicator in subarachnoid hemorrhage. Hippocampal tissue from subarachnoid hemorrhage mice showed reduced expression of acetyl-coenzyme A synthetase-2 and abnormalities in the expression of proteins related to ribosomes, energy metabolism, and cellular signal transduction. This study confirmed telomere shortening in the brain and metabolic disturbances in the hippocampi of subarachnoid hemorrhage mice. Thus, telomere length is a predictive marker for long-term impairment of cognitive function in mice following experimental subarachnoid hemorrhage.

## Graphical abstract

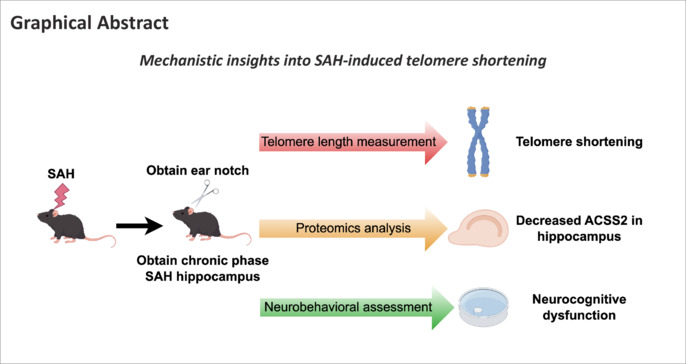

## Introduction

Subarachnoid hemorrhage (SAH), a subtype of stroke, accounted for 9.7% of all patients with stroke in 2019, making it the third most common stroke type. The poor prognosis of patients with SAH represents a substantial burden on the global economy (GBD 2019 Stroke Collaborators, 2021). The direct toxic effect of heme on nerve cells in SAH induces iron death, oxidative stress, inflammatory reaction, cerebral vasospasm (CVS), delayed cerebral ischemia (DCI), and other complications, which seriously impair nerve function in patients and lead to poor long-term prognosis. Patients with SAH exhibit impaired function, struggle with self-care, experience long-term immobility, and may even face coma or death (Claassen and Park, 2022).

Recovery of neurological function after SAH is a dynamic process. Studies have demonstrated that, within a period of 6 months to 1 year following discharge, only 22.6% of patients with SAH exhibit neurological function improvement, as measured by the modified Rankin scale (mRS) score, and patients with an mRS score greater than 3 rarely improve (Hammer et al., 2020). These findings suggest that, particularly for patients with SAH with severe symptoms, the damage to their neurological function is long-term and enduring, making it challenging to achieve improvement through rehabilitation. Patients with a poor long-term prognosis exhibit not only impaired autonomous function, but also cognitive impairment. This impairment manifests in various ways, such as memory decline, anxiety, depression, and other neuropsychological changes (Danala et al., 2022). A follow-up study has demonstrated that an increasing proportion of patients exhibits cognitive impairment, as assessed by the Montreal Cognitive Assessment Scale (MoCA) criteria, from 3 months to 1 year after SAH (Danala et al., 2022). Whether the long-term cognitive impairment of patients with SAH represents persistent neurological impairment after the acute stage of SAH and the mechanism underlying this phenomenon require further investigation.

Telomeres are repetitive, non-coding DNA sequences located at the terminal regions of linear eukaryotic chromosomes (Revy et al., 2023; Ying et al., 2025). They serve as biomarkers that are closely associated with aging (Rossiello et al., 2022), and play four crucial functions within cells. First, they safeguard vital genetic information from erosion during DNA replication. Second, they shield DNA strands from damage that could trigger apoptosis. Third, they bind and recruit proteins essential for DNA repair processes. Finally, they serve as a mitotic clock, providing insight into the proliferation history of individual cells (Eitan et al., 2014). Maintaining optimal telomere length requires a delicate and intricate balance between telomere elongation mechanisms and telomere shortening processes (Revy et al., 2023). Telomeres can undergo shortening not only through cell division and replication, but also in response to a variety of other factors. Tissues with high metabolic rates and limited regenerative capacity, such as neurons, are particularly vulnerable to oxidative stress–induced damage and subsequent telomere shortening (Klapper et al., 2001; Smith et al., 2013). The oxidative damage inflicted on telomeric DNA is believed to contribute to premature aging by accelerating telomere attrition. Recent research has shown that oxidative stress can not only lead to telomere shortening but also cause oxidative base damage within telomeric regions. One commonly occurring DNA lesion is 8-oxo-guanine (8-oxoG), which has been identified in telomeres in human fibroblasts and epithelial cells (Barnes et al., 2022). Furthermore, the presence of telomeric 8-oxoG is sufficient to activate the p53 signaling pathway, which is involved in aging. Telomeric 8-oxoG triggers the ataxia telangiectasia mutated (ATM) and ataxia telangiectasia and Rad3-related (ATR) signaling pathways, leading to the accumulation of markers indicating telomere dysfunction in actively replicating cells, rather than dormant cells (d’Adda di Fagagna et al., 2003; Sfeir and de Lange, 2012). This results in fragile sites within telomeres and hinders DNA replication (Barnes et al., 2022). Consequently, oxidative stress can accelerate aging by expediting telomere shortening and promoting oxidative base damage.

Previous studies established the pivotal role of oxidative stress in neurological impairment resulting from SAH (Lauzier et al., 2023; Zhang et al., 2024). However, little is known about the intricate pathogenesis of SAH, particularly the mechanisms that contribute to long-term cognitive dysfunction. Specifically, it remains unclear whether oxidative stress–induced telomere attrition instigates neurocognitive aging-like phenomena during the chronic phase of SAH. Therefore, the primary objective of this study was to assess the utility of telomere length as a biomarker for predicting long-term prognosis in SAH. Additionally, we investigated the mechanisms underlying adverse long-term SAH outcomes, with a particular focus on discerning whether telomere loss induced by oxidative stress contributes to this phenomenon.

## Methods

### Animals

For this study, 40 male C57BL/6J mice (6–8 weeks old, body weight 24 ± 0.29 g) were used. The physiological state of female mice is significantly influenced by cyclic fluctuations of estrogen and progesterone, which may lead to increased variability in experimental results. Male mice, which have more stable hormone levels, are therefore more commonly used in experiments where reducing variability is essential (Beery and Zucker, 2011). The mice were acclimated to an environment with constant temperature (21 ± 1°C) and humidity (55% ± 5%) and a 12-hour light/dark cycle for one week prior to surgery. During this period, they had unrestricted access to water and food. The mice was randomly divided into three groups: the SAH group, Sham group, and Control group. All experiments were conducted in accordance with the National Institutes of Health Guide for the Care and Use of Laboratory Animals (8^th^ ed., National Research Council, 2011). All animals procedures were carried out in strict accordance with the relevant regulations regarding animal ethics and laboratory animal protection of the Laboratory Animal Center of Zhejiang University, and all animal experiments were approved by the Ethical Review Committee of Laboratory Animal Welfare of Zhejiang University (approval No. ZJU20230290) and reported in accordance with the ARRIVE guidelines (Animal Research: Reporting of *In Vivo* Experiments; Percie du Sert et al., 2020).

### Establishment of a mouse model of subarachnoid hemorrhage

An intravascular puncture model of SAH was established in mice, as previously described (Liu et al., 2021). Briefly, the mice were anesthetized with 2% isoflurane (RWD Life Science, Shenzhen, Guangdong, China) via inhalation followed by intraperitoneal injection of 1% pentobarbital sodium (0.05 mg/g body weight) to maintain anesthesia. The depth of anesthesia was confirmed by the absence of response to pain stimuli (such as toe pinching). A sharp 5-0 monofilament nylon suture was insert into the left carotid artery from the external carotid artery and common carotid bifurcation. The suture was advanced until the bifurcation of the anterior and middle cerebral arteries. The suture was further advanced an additional 3 mm to puncture the vessel. The suture was held in place for a duration of 10 seconds prior to being withdrawn. Observable signs such as muscle tremors, altered respiratory patterns, cardiac rhythm variations, and urinary incontinence indicated successful model establishment (Liu et al., 2021). After the surgery, the mice were transferred to a controlled environment within a temperature-regulated chamber to facilitate their gradual recovery until they fully regained consciousness. The mice in the sham group underwent the same procedure as those in the SAH group, without puncturing the left internal carotid artery. The control group mice did not undergo any surgical intervention.

Following complete recovery, the mice in all three groups (SAH group, sham group, control group) were housed under standard conditions for 24 hours. Mice exhibiting evident contralateral limb paralysis were excluded from the study. Some mice in the SAH group exhibited only minimal or extremely limited bleeding. Out of the initial twenty SAH mice, three without significant neurological deficits (score > 15 on the modified Garcia scale) 24–48 hours after the operation were excluded. Four mice in the SAH group did not survive past 48 hours (resulting in a mortality rate of 20%), although none of them displayed contralateral limb paralysis. Thus, 13 mice were ultimately included in the SAH group, and, along with the 10 mice in the Sham group and the 10 mice in the Control group, were housed under identical conditions for further observations.

### Assessment of cognitive function alterations following subarachnoid hemorrhage via neurobehavioral testing

**Figure 1:**
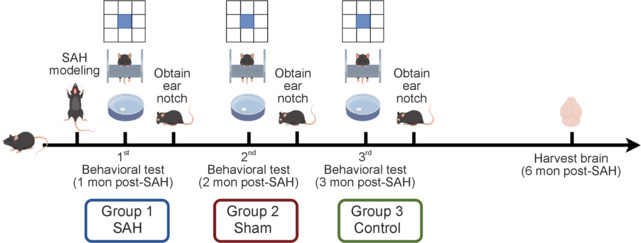
Experimental timeline. The rotarod test, Morris water maze test, and open field test were conducted three times. Ear notch epithelial samples were collected and preserved at the indicated time points after each round of behavioral testing. The brains of the mice from each group were collected after 6 months of SAH modeling. SAH: Subarachnoid hemorrhage.

In the clinical setting, the period spanning 24 to 72 hours following SAH is commonly referred to as the early brain injury phase, while the timeframe from 72 hours to 2 weeks is characterized by a heightened risk of cerebral vasospasm. Notably, cognitive impairment typically becomes evident after the initial 2-week period following SAH (Macdonald and Schweizer, 2017; Hoh et al., 2023). Consequently, the present study aimed to evaluate long-term alterations in cognitive function in mice following SAH. We conducted behavioral assessments at monthly intervals starting 1 month post-surgery. These assessments encompassed various aspects of neurocognitive function, including motor skills, memory, and levels of anxiety and depression across the different experimental groups. Three behavioral tests were administered on separate experimental days (**Figure 1**), as described, behavioral tests were conducted at monthly intervals, with specific experimental protocols and methodologies described in detail below.

#### Rotarod test

This experiment was conducted using a Panlab Rotating Rod Instrument (Rotarod Model LEB205, Panlab S.L.U, Spain), as previously described (Song et al., 2020). The experimental protocol consisted of a training phase spanning days 1 to 3, followed by formal testing on day 4. Before each experimental session, the mice underwent a 1-minute adaptation period on the stationary rod. During the three days of training, the rod was programmed to rotate at consistent speeds of 4 revolutions per minute (RPM), 8 RPM, and 12 RPM. Each mouse underwent three daily training sessions, lasting 1 minute each, with a 1-minute rest interval between sessions. On the 4^th^ day, the testing phase involved a uniform acceleration exercise. The rotational speed of the rod was gradually increased from 4 RPM to 40 RPM over a period of 300 seconds. Each mouse underwent three testing trials, and the maximum time spent on the rod before falling (in seconds) was recorded for analysis.

#### Morris water maze

The Morris water maze experiment was conducted over 6 days, consisting of 5 training days (day 1 to day 5) and one testing day (day 6), to evaluate the spatial and long-term memory capabilities of the mice. The experiment utilized a circular pool with a diameter of 1.5 meters, equipped with additional visual aids. The water temperature was maintained at 22–25°C, and the pool water was rendered opaque white using titanium dioxide powder. Before each trial, the pool water was thoroughly mixed to ensure even dispersion of the dye. Mouse behavior was analyzed using Watermaze Analysis Software (Version 4.07, Actimerics software, Wilmette, IL, USA). The circular pool was divided into four quadrants. An escape platform, measuring 10 cm in diameter, was positioned in the center of quadrant III, about 1–2 cm below the water surface, rendering it invisible to the naked eye. During the training sessions from day 1 to day 5, the mice were introduced to the water four times, each time starting from a different quadrant while facing the center. The order of quadrants was randomized. If a mouse successfully reached the platform within 60 seconds and remained on it for at least 10 seconds, it was considered to have found the platform, and the trial ended automatically. The time taken by each mouse to locate the platform in each trial was recorded as the latency. In cases where a mouse failed to locate the escape platform within 60 seconds, it was manually guided to the platform and allowed to remain on it for 10 seconds. Following each training session, the mice were dried and placed in a dry incubator to facilitate fur drying. The latency periods for the mice to find the platform after entering the water in quadrant I on day 1 to day 5 were recorded and compared to evaluate spatial memory abilities, which reflect learning and memory.

On the test day, the platform was removed, and each mouse was placed in the water in quadrant I and allowed to swim freely 60 seconds. Parameters such as the latency to the original escape platform location, activity time (% of time spent swimming), and distance traveled in quadrant III were recorded and analyzed to assess long-term memory. The mice were dried and placed in a dry incubator for rest after each test session.

#### Open field test

A white cube-shaped open field arena measuring 45 cm on each side was utilized to conduct behavioral assessments. Mouse activity was observed and analyzed using Anymaze analysis software (version 6.03, Steolting software, Wood Dale, IL, USA). The open field box was divided into nine equal parts, consisting of a central area and surrounding areas. For each experiment, mice were gently placed into the central area and given 300 seconds to freely explore their surroundings. The baseline activity of mice was assessed by analyzing parameters such as distance and average speed. Mice typically exhibit a preference for staying near the walls of the open field box. However, due to their innate curiosity, they gradually explore the central area. Extended periods spent in the surrounding areas and corners of the open field box, accompanied by reduced exploration of the central region, are often associated with increased levels of anxiety and depression. To evaluate the anxiety and depression levels of the mice, we analyzed several key indices, including distance traveled, activity time ratio, center entries per total entries, and time spent mobile within the surrounding and central areas.

#### Quantification of cognitive function severity

To assess the extent of cognitive impairment in experimental mice, we used a comprehensive cognitive function scoring system (Hehar and Mychasiuk, 2016; **Table 1**). This scoring system was standardized based on the average performance score of the Control group as the reference point. Scores exceeded this baseline were assigned a value of 1, while scores falling below it were assigned a value of 0. Scores of 2/6 or less were classified as poor performance, scores ranging from 3/6 to 4/6 were categorized as moderate performance, and scores of 5/6 or higher were classified as good performance. This scoring system was employed to evaluate the performance levels of the animals in the study.

**Table 1:** Cognitive function scores based on behavioral tests

Behavioral test	Evaluation content	Test content	Score
Rotarod test	Active ability	Maximum activity time	1/0
Morris Water Maze	Spatial memory	Training day latency	1/0
	Long-term memory	Test day latency	1/0
		Test day durance	1/0
Open field test	Anxiety and depression level	Immobilize time	1/0
		Number of center explorations	1/0
Total score	0/6 ~ 6/6		

The behavioral assessment items of the SAH group and sham group, which were better than those of the control group, were scored as 1; otherwise, they were scored as 0.

### Quantitative polymerase chain reaction analysis of relative telomere length

To minimize the potential interference of stress during collection of ear notch samples from the mice, we collected the samples following completion of behavioral experiments. At 6 months, ear notch tissue was collected before the mouse brains were harvested. At each sampling time point, a small section of ear notch epithelial tissue, measuring approximately 2 mm × 3 mm, was collected. These tissue samples were then meticulously placed into Eppendorf (EP) tubes and subsequently stored at –80°C. At 6 months post-surgery, anesthesia was induced in the mice with 2% isoflurane, the mice were sacrificed, and the whole brain was quickly removed. The prefrontal cortex and hippocampus were meticulously dissected and sliced into smaller fragments, which were promptly stored at –80°C to preserve their integrity and viability.

Genomic DNA (gDNA) was extracted from the ear notch and brain tissue samples using a TIANamp Genomic DNA Kit (TIANGEN BIOTECH, Beijing, China) according to the manufacturer’s instructions. Subsequently, the quality and concentration of the extracted gDNA were assessed using a NanoDrop One (Thermo Fisher Scientific, Waltham, MA, USA). To facilitate telomere analysis, the samples were uniformly diluted to 10 ng/μL. Primers targeting both the telomere gene (Tel) and the internal reference gene (36B4) were meticulously designed based on Cawthon’s previous research (Cawthon, 2002) and data from the National Biotechnology Information Center. The primers were prepared at a concentration of 10 μM in TE buffer. Detailed information regarding primer sequences and thermocycling conditions can be found in **Table 2**.

**Table 2:** Primer and cycling information for qPCR for relative telomere length analysis

Gene (symbol)	Primer sequence	Temperature (°C)	Cycling parameters
Telomere (Tel)	F: CGG TTT GTT TGG GTT TGG GTT TGG GTT TGG GTT TGG GTT R: GGC TTG CCT TAC CCT TAC CCT TAC CCT TAC CCT TAC CCT	54	1 cycle 95°C 15 min 40 cycles 95°C 15 s 40 cycle 55°C 20 s 40 cycles 72°C 20 s + Melt curve
Acidic ribosomal phosphoprotein P0 (36B4)	F: AGA TTC GGG ATA TGC TGT TGG C R: TCG GGT CCT AGA CCA GTG TTC	62	1 cycle 95°C 15 min 40 cycles 95°C 15 s 40 cycle 55°C 20 s 40 cycles 72°C 20 s + Melt curve

F: Forward; qPCR: quantitative polymerase chain reaction; R: reverse.

Quantitative polymerase chain reaction (qPCR) reactions were conducted in triplicate in a 384-well plate. Each qPCR reaction consisted of 1 μL of gDNA in a total volume of 10 μL, used SuperReal PreMix Plus (SYBR Green) (TIANGEN BIOTECH), and was performed on a LightCycler 480 instrument (Roche, Basel, Switzerland). To rule out reagent contamination, a no-template control (NTC) was included for each thermocycling condition. To determine telomere length, the ratio of telomere gene expression level to the expression level of the internal reference gene (36B4) was calculated using the equation [2^Ct(telomere)^/2^Ct(36B4)^]^–1^ = 2^–ΔCt^, as described by Cawthon (Cawthon, 2002). The relative telomere length was subsequently calculated utilizing the equation 2^–(ΔCtsample 1–ΔCtsample 2)^ = 2^–ΔΔCt^, enabling determination of the telomere length ratio for mice within each experimental group relative to the telomere length of the corresponding control mice. gDNA from a separate set of experimental mice was employed as a reference control (RC). The telomere length of the mice in each group was compared to the telomere length of the mice in the RC group.

### Brain hippocampus tissue proteomic profiling

Peptides were extracted from the hippocampal tissue of SAH mice and age-matched C57BL/6J mice. After desalting, the peptides were subjected to mass spectrometry analysis. For each sample, 2 g of total peptide was separated and analyzed with a nano UPLC (EASY-nLC1200) coupled to a Q Exactive HFX Orbitrap instrument (Thermo Fisher Scientific) with a nano-electrospray ion source. Separation was performed using a reversed phase column (100 μm ID × 15 cm, Reprosil-Pur 120 C18AQ, 1.9 μm, Dr. Maisch, Beim Brückle 14, 72119 Ammerbuch-Entringen, Germany). The mobile phases were H_2_O with 0.1% formic acid (FA), 2% acetonitrile (ACN) (phase A), and 80% ACN, 0.1% FA (phase B). Sample separation was executed with a 120-minute gradient at a 300 nL/min flow rate. Gradient B: 25% for 2 minutes, 522% for 88 minutes, 2245% for 26 minutes, 4595% for 2 minutes, 95% for 2 minutes. The peptides extracted from mouse hippocampal tissue were subjected to mass spectrometry by Shanghai Biotree Biotech Co., Ltd.

Data-dependent acquisition (DDA) was performed in profile and positive mode with an Orbitrap analyzer at a resolution of 120,000 (@200 m/z) and a m/z range of 3501600 for MS1; for MS2, the resolution was set to 15,000 with a dynamic first mass. The automatic gain control (AGC) target for MS1 was set to 3E6 with max IT 50 ms, and 1E5 for MS2 with max IT 110 ms. The top 20 most intense ions were fragmented by HCD with a normalized collision energy (NCE) of 27% and an isolation window of 1.2 m/z. The dynamic exclusion time window was 45 seconds. Single charged peaks and peaks with a charge exceeding 6 were excluded.

The vendor’s raw MS files were processed using Proteome Discoverer (PD) software (Version 2.4.0.305) and the built-in Sequest HT search engine. MS spectra lists were searched against their species-level UniProt FASTA databases (uniprot-Mus musculus-10090-2022-11. fasta), with carbamidomethyl [C] as a fixed modification, and oxidation (M) and acetyl (protein N-term) as variable modifications. Trypsin was employed as the protease, allowing for a maximum of two missed cleavage events. The false discovery rate (FDR) was set to 0.01 for both PSM and peptide levels. Peptide identification was performed with an initial precursor mass deviation of up to 10 ppm and a fragment mass deviation of 0.02Da. A unique peptide and the Razor peptide were used for protein quantification and total peptide amount for normalization.

### Statistical analysis

No statistical methods were used to predetermine sample sizes; however, our sample sizes are similar to those reported in a previous publication (Hehar and Mychasiuk, 2016). To ensure the repeatability of measurements, all telomere length analyses and behavioral tests were conducted by two independent, trained observers. Each observer performed the measurements in a blinded manner to avoid bias. To assess inter-observer reliability, a subset of samples (e.g., 20%) was randomly selected and analyzed by both observers. The consistency of the results was evaluated using the intraclass correlation coefficient (ICC), with values greater than 0.75 considered indicative of good reliability.

Statistical analysis was carried out using GraphPad Prism (version 10.0.2 for Mac, GraphPad Software, San Diego, CA, USA, www.graphpad.com). To assess the disparities in cognitive function and telomere length across the various groups, one-way analysis of variance was conducted, followed by Bonferroni’s multiple comparisons test. Furthermore, Pearson’s correlation test was employed to gauge the strength of the correlation between prefrontal cortex tissue and hippocampus relative telomere length and the severity of cognitive function impairment. A significance level of *P* < 0.05 was considered statistically significant.

## Results

### Prolonged deterioration in mouse cognitive function following subarachnoid hemorrhage

#### Long-term decline in motor ability in subarachnoid hemorrhage mice, as assessed by rotarod test

**Figure 2:**
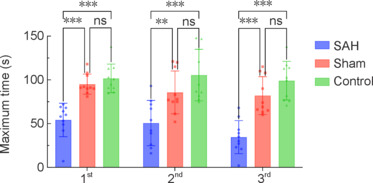
The rotarod experiments reveal the continued impairment of motor ability in SAH mice. All experiments were repeated three times. Data are presented as mean ± SD. **P < 0.01, ***P < 0.001. *n* = 10 mice. ns: Not significant; SAH: subarachnoid hemorrhage.

In all three rotarod tests, the mice in the SAH group consistently exhibited significantly shorter maximum activity times compared with the mice in both the Sham group and the control group (*P* < 0.001; **Figure 2**). Conversely, no significant differences were observed between the sham group and the control group. These findings provide evidence of a persistent impairment in mouse motor abilities following SAH, highlighting the detrimental impact that SAH has on their physical performance.

#### Enduring impairment of both long-term memory and spatial memory in subarachnoid hemorrhage mice, as assessed by the Morris water maze test

**Figure 3:**
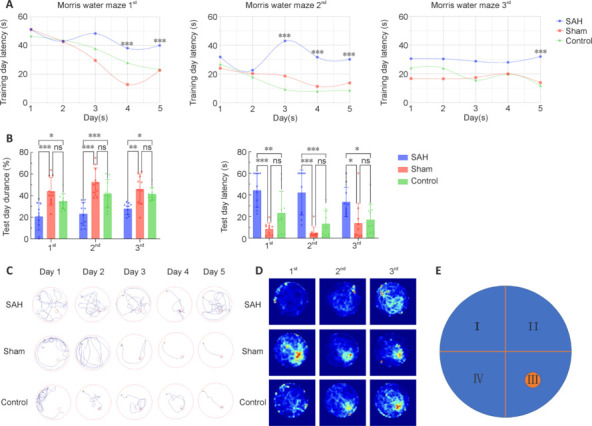
The results of three trials of the Morris water maze. (A) The results of the training days suggest that spatial memory impairment persists in SAH mice over the long term. (B) The results of the test days indicate that long-term memory impairment continues to exist in SAH mice. (C) The activity path map during the initial training day of the Morris water maze test suggested neurological dysfunction and impaired learning and memory abilities in SAH mice. (D) Activity heatmaps from the three Morris water maze test days show that SAH mice spent significantly less time in the target quadrant (quadrant III) compared to the sham and control groups, indicating persistent impairment in their long-term memory ability. (E) Diagram illustrating the division of the Morris water maze apparatus. All experiments were repeated three times. Data are presented as mean ± SD. *P < 0.05, **P < 0.01, ***P < 0.001 (*vs*. control in A). *n* = 10 mice. ns: Not significant; SAH: subarachnoid hemorrhage.

During the training period, the mice in the SAH group exhibited a significantly longer latency in finding the escape platform than mice in the control group (*P* < 0.001; **Figure 3A**). The latency in the training phase is associated with spatial memory capacity. Our findings suggest that spatial memory was significantly impaired in SAH mice compared with the sham and control groups (*P* < 0.001, **Figure 3A**), indicating long-term cognitive deficits in SAH mice. Furthermore, the mice in the SAH group exhibited more disordered activity paths than the mice in the other two groups during training (**Figure 3C**), indicating neurocognitive impairment. The SAH mice showed a slower rate of learning and less memory of the escape platform location compared with the Sham and Control mice.

The results from the Morris water maze test days can be analyzed in terms latency, which is the time taken by each mouse to reach the platform, and the proportion of time spent in the quadrant that contained the escape platform. These parameters reflect the long-term memory capacity of the mice. We found that the SAH mice exhibited significantly longer latencies than the sham and control mice (**Figure 3B**). Additionally, the mice in the SAH group spent significantly less time in the target quadrant relative to the mice in the sham and control groups in all three tests (*P* < 0.001; **Figure 3B**). Moreover, the activity heatmaps from the three tests showed that both the sham and control mice displayed notably longer activity durations within the target quadrant compared with the SAH group (**Figure 3D**). These results collectively indicate a marked decline in long-term memory capacity in the SAH group in comparison with the control group.

#### Continuous increase in anxiety and depression levels in subarachnoid hemorrhage mice, as determined by the open field test

In the open field test, the distance covered by each mouse in a single trial is considered its baseline activity level. Elevated levels of anxiety and depression in mice are associated with a tendency to linger in proximity to the walls and corners of the open field box, accompanied by prolonged periods of immobility and reduced exploration of the central open area. Consequently, the time spent in the central area and the duration of immobility serve as markers of anxiety and depression in mice. These psychological states exhibit an inverse relationship with activity within the central area, while concurrently demonstrating a direct correlation with the frequency and duration of immobile periods.

**Figure 4:**
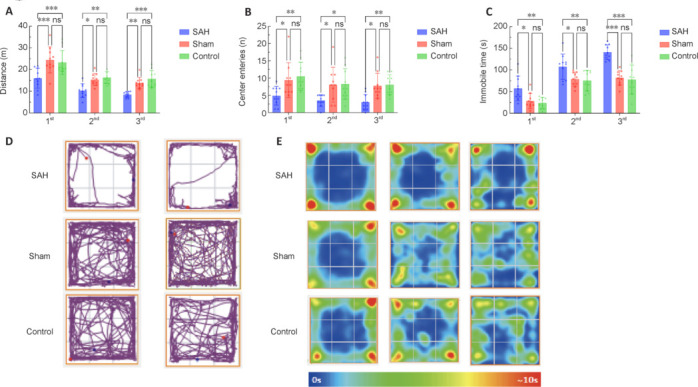
The results of the open field test revealed elevated anxiety and depression levels among SAH mice. (A) SAH mice exhibited prolonged decrease in basal activity. (B) Reduction in the exploratory behavior of the central region in SAH mice. (C) Sustained increase in immobile time in SAH mice. (D) The pathway map generated from the open field test indicated a decrease in the baseline activities of SAH mice, along with reduced exploration of the central areas. (E) The heatmap of the open field test revealed that SAH mice spent more time in the corners of the open field box compared to the other groups. All experiments were repeated three times. Data are presented as mean ± SD. *P < 0.05, **P < 0.01, ***P < 0.001. *n* = 10 mice. ns: Not significant; SAH: subarachnoid hemorrhage.

We observed a significant decrease in the baseline activity levels of the SAH mice compared with the sham and control mice (**Figure 4A**). Consistently, significantly longer periods of immobility were observed in the SAH group compared with the sham and control groups (*P* < 0.05; **Figure 4C**). Furthermore, there was a substantial decrease in the number of SAH group entries into the central area compared with the sham and control groups (*P* < 0.05; **Figure 4B**). This decrease in exploration of the central area implies reduced curiosity and heightened anxiety and depression levels in the SAH group. Additionally, assessment of typical activity path maps (**Figure 4D**) and activity heatmaps (**Figure 4E**) during the three open field tests showed that the mice in the SAH group predominantly inhabited the peripheral zones, specifically in close proximity to the walls and corners of the open field box, indicative of a substantial reduction in their interaction with the central area and suggesting a significant decrease in their exploratory drive, a key factor in understanding their neurocognitive function.

#### Quantification of cognitive function

**Figure 5:**
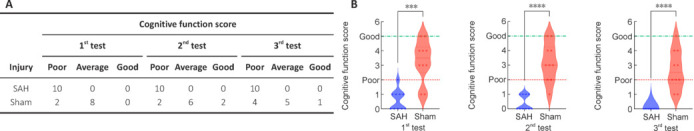
Description of long-term cognitive function in the SAH and sham groups. (A) Rating results of three behavioral tests in the sham and control groups. (B) Significant difference between three behavioral tests of mice in the sham and control groups. All experiments were repeated three times, ***P < 0.001, ****P < 0.0001. Number of animals = 10. ns: Not significant; SAH: subarachnoid hemorrhage.

Mice in both the SAH and Sham groups were classified into three performance categories (poor, average, and good) based on the scoring criteria described in **Table 1**. The cognitive function of SAH mice remained impaired across all three behavioral evaluations (**Figure 5**). Compared with control mice, SAH mice showed poor performance in every neurobehavioral test. Sham mice showed a slight improvement in the second test compared with the first test, which may be related to the learning and memory formed by repeated training, and may reflect differences in learning and memory between Sham mice and SAH mice.

### Telomere length and cognitive performance in subarachnoid hemorrhage mice

**Figure 6:**
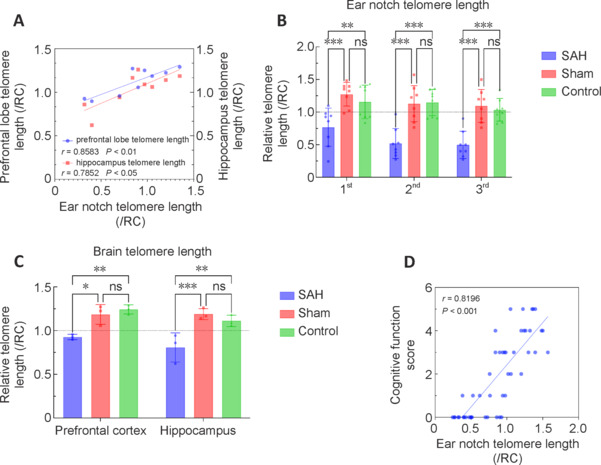
Comparisons of telomere length and the correlation between telomere length and cognitive function in mice following SAH. (A) Relative telomere length in 6-month-old ear notch skin cells demonstrated a significant positive correlation with relative telomere length extracted from prefrontal cortex tissue (*r* = 0.8583, *P* < 0.01) and the hippocampus (*r* = 0.7852, *P* < 0.05) within the same animals (*n* = 9). (B) Telomere length analysis of ear notch skin cells before each behavioral test (*n* = 8). (C) Comparison of telomere length in the prefrontal cortex and hippocampus of mice in each group after 6 months of modeling (*n* = 3). (D) All cognitive function scores and corresponding telomere lengths of SAH and Sham mice in three behavioral tests were analyzed synthetically, and telomere length significantly and positively predicted the neurofunction score of the mice after SAH (*r* = 0.8196, *P* < 0.001). All experiments were repeated three times. *P < 0.05, **P < 0.01, ***P < 0.001. ns: Not significant; SAH: subarachnoid hemorrhage. RC stands for relative control, which refers to the average value of the control group. The relative telomere length is normalized by dividing the results from the SAH, Sham, and Control groups by the average value of the control group. RC: Reference control.

The correlation between telomere length in genomic DNA isolated from ear notch skin cells and prefrontal cortex and hippocampus telomere length was validated in both normal and SAH mice (**Figure 6A**). A significant reduction in total telomere length in skin DNA was observed in SAH mice after all three behavioral tests (**Figure 6B**). Furthermore, we found a significant decrease in telomere length in prefrontal cortex and hippocampus DNA among SAH mice (**Figure 6C**). Correlation analysis revealed a strong association between telomere length and cognitive function in the SAH and Sham groups (**Figure 6D**).

### Proteomics analysis of the hippocampus in subarachnoid hemorrhage mice

**Figure 7:**
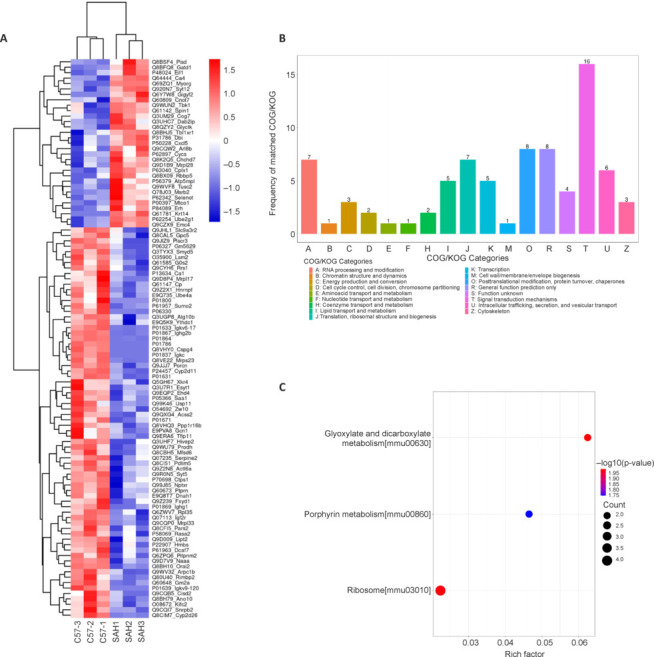
Proteomic analysis of the hippocampus in mice following SAH. (A) EuKaryotic Orthologous Groups (KOG) analysis revealed that proteins related to signal transduction functions were predominantly among the differentially expressed proteins in SAH mice compared with normal mice. (B) Bubble chart depicting notable differences in glyoxylate and dicarboxylate metabolism and ribosome function in the hippocampal tissues of SAH mice compared with those of normal mice. (C) Hierarchical cluster analysis further emphasized the differences in protein expression within the hippocampus between SAH mice and normal mice and revealed a significant decrease in the expression of ACSS2 in SAH mice. *n* = 3 mice. ACSS2: Acetyl-coenzyme A synthetase-2; SAH: subarachnoid hemorrhage.

Proteomic analysis was performed on the bilateral hippocampus tissue of mice 6 months after SAH. The results revealed significant disparities in protein expression between SAH mice and control mice (**Figure 7A**). Hierarchical clustering showed distinct patterns of protein grouping between SAH mice and control mice. Specifically, the expression level of acetyl-CoA synthetase 2 (ACSS2), which is associated with of acetyl-coenzyme A synthesis and expression, as well as modulation of histone acetylation and synaptic transmission (Mews et al., 2017), was significantly decreased in SAH mice. EuKaryotic Orthologous Groups (KOG) analysis (**Figure 7B**) revealed substantial differences in the expression of proteins related to signal transduction between chronic SAH mice and their control counterparts, corroborating the findings from the hierarchical clustering analysis.

Furthermore, a KEEG annotation enrichment analysis revealed notable disparities in glyoxylate and dicarboxylic acid metabolism within the hippocampus of SAH mice compared with control mice (**Figure 7C**). These differences were linked to acetyl coenzyme A synthesis, which is associated with ACSS2 and influences glucose and energy metabolism in the nervous system. This finding indicates that ACSS2 not only influences synaptic plasticity but also contributes to dysfunctional tricarboxylic acid cycling in the hippocampus of SAH mice. Snieckute et al. (2023) found that ribosomal damage caused by reactive oxygen species (ROS) can lead to unnecessary metabolic maladjustment in obesity and aging, which corresponds to the significant difference in the expression of ribosomal-related proteins in SAH mice in this study. Abnormal metabolism in the nervous system caused by ROS-induced ribosome damage during SAH may also contribute to nervous system dysfunction. These disruptions in energy metabolism may promote the cognitive dysfunction observed in SAH mice.

## Discussion

Here we introduce the concept of chronic brain injury in SAH and discuss its implications for future research. Following SAH, even patients with mild cases frequently experience varying degrees of long-term cognitive impairment, despite rehabilitation efforts. The latest guidance highlights the importance of assessing cognitive function during SAH rehabilitation (Hoh et al., 2023). Currently assessment methods rely on subjective scales, such as the Montreal Cognitive Scale (Cornea et al., 2022). Although functional magnetic resonance imaging and event-related potential have been used to assess cognitive functional changes in conditions such as stroke and Alzheimer’s disease (Ellmore et al., 2013; Wang et al., 2022; Qin et al., 2023), there is a lack of practical approaches and objective indicators to determine long-term cognitive prognosis in patients with SAH.

Telomere length shortening is regarded as an aging indicator (López-Otín et al., 2023). Factors like DNA replication, inflammation, and oxidative stress contribute to telomere length reduction (Smith et al., 2013; Ahmed and Lingner, 2018; Barnes et al., 2019). Notably, neurons exhibit low levels of telomerase gene expression, making it difficult to restore telomere length once it diminishes, leading to persistent shortening. Oxidative stress plays a significant role in early brain injury following SAH (Rass and Helbok, 2019). Given that SAH surpasses the body’s oxidative stress threshold in its early stages, oxidative stress may be a primary contributor to telomere shortening in mouse models of SAH. Considering the link between telomere shortening and aging, the long-term cognitive deficits that we observed in SAH mice may resemble aging-like symptoms, potentially leading to neurological decline and cognitive impairment.

Previous studies have investigated the relationship between telomere shortening and neurocognitive function impairment in rats with traumatic brain injury (Hehar and Mychasiuk, 2016). Telomere length in brain tissue can be indirectly assessed by measuring telomere length in skin cells (Hehar and Mychasiuk, 2016). In this study, the telomere length in brain tissue from SAH mice was found to be shorter than that in normal mice of the same age, as assessed by qPCR. Additionally, we confirmed the relationship between telomere length in ear skin cells and brain tissue, suggesting that telomere shortening in brain tissue occurs in the early and late stages disease in SAH mice. Utilizing various behavioral evaluation methods, we assessed long-term changes in neurocognitive function among SAH mice in the chronic stage of disease. These assessments revealed enduring neurocognitive impairments in SAH mice, encompassing aspects such as motor skills, memory capacity, anxiety, and depression. Furthermore, cognitive function in both the SAH and Sham groups were quantified using a standardized scale, providing evidence of a correlation between telomere length and cognitive function in mice. Previous studies have shown that inflammatory factors and oxidative stress–related indices such as NOX4 are related to SAH prognosis and can be used as prognostic biomarkers of SAH (Pan et al., 2021; Luo et al., 2022). Chronic inflammation and elevated oxidative stress levels lead to excessive DNA damage, particularly in telomeres, due to their high susceptibility to ROS (Sampson et al., 2006; Houben et al., 2008). This accelerates telomere attrition, driving cellular senescence. Senescent cells with critically shortened telomeres often adopt a senescence-associated secretory phenotype (SASP), characterized by the secretion of pro-inflammatory cytokines, growth factors, and proteases (Wang et al., 2024). The SASP may further exacerbate local and systemic inflammation, potentially creating a feedback loop that amplifies oxidative stress and telomere damage. This dynamic interplay among telomere shortening, SASP, and oxidative stress is hypothesized to be particularly relevant in SAH pathology, where persistent inflammation and oxidative stress could contribute to worse neurological outcomes.

The long-term prognosis of patients SAH exhibits alarming parallels with brain aging. Our study revealed substantial cognitive impairment in SAH mice, along with telomere shortening in the hippocampus, a recognized biomarker of aging (Moqri et al., 2023), which suggests potential brain aging in these mice. Additionally, our proteomic analysis unveiled significant metabolic dysfunction, particularly a reduction in ACSS2 expression. This raises the intriguing question of whether the cognitive deficits observed in the chronic stage of SAH could be interpreted as a secondary manifestation of brain aging–like processes.

Pathological aging poses a threat to various cognitive functions, including consciousness, emotion, memory, language, and sensorimotor abilities. It is characterized by the development of neurodegenerative disorders and cognitive impairment (St Sauver et al., 2015). Aging is associated with epigenetic alterations, predominantly driven by DNA methylation, which in turn lead to biochemical changes, including telomere attrition, genomic instability, protein imbalances, and lipid imbalances. These alterations subsequently lead to a series of cellular transformations, including mitochondrial dysfunction. Ultimately, this results in cognitive decline, a diminished capacity for self-care, and manifestation of clinical disorders (Higgins-Chen et al., 2021). The acute phase of SAH is characterized by overwhelming oxidative stress, which has been postulated as a potential trigger for telomere attrition. This acceleration of brain aging exerts profound effects on the normal energy metabolism and signal transduction within the central nerve system (Higgins-Chen et al., 2021).

Targeting oxidative stress is a promising therapeutic strategy for mitigating SAH-induced damage. Central to this approach is activation of the nuclear factor erythroid 2-related factor 2 (Nrf2) signaling pathway. Nrf2 is a transcription factor that regulates the expression of a broad range of antioxidant and detoxification enzymes, including heme oxygenase-1 (HO-1), superoxide dismutase (SOD), and glutathione peroxidase. These enzymes play crucial roles in neutralizing ROS, maintaining redox balance, and protecting against oxidative injury. Pharmacological agents that enhance Nrf2 activity have demonstrated efficacy in preclinical models of SAH by alleviating oxidative damage, reducing neuroinflammation, and improving neurological outcomes (Cahill and Zhang, 2009; Zhao et al., 2016).

In addition to its antioxidative functions, Nrf2 also regulates genes involved in cellular repair and metabolism, contributing to the restoration of neuronal and vascular integrity. Compounds such as sulforaphane and curcumin have shown potential in activating the Nrf2 pathway and mitigating SAH-related oxidative stress (Zhao et al., 2016; Xu et al., 2025). By enhancing the expression of antioxidant enzymes in the early phase of SAH, these therapies can reduce oxidative damage, improve cerebral blood flow, and support neurovascular recovery.

Histone acetylation plays a crucial role in memory formation, coordinating chromatin remodeling in various brain regions associated with learning and memory, particularly the hippocampus (Mews et al., 2017). In the hippocampus of aged mice, impaired histone H2B/H4 acetylation during learning hinders the initiation of gene expression related to memory consolidation (Bousiges et al., 2010; Peleg et al., 2010). Neuronal ACSS2 plays a dual role, influencing both acetyl-coenzyme A synthesis and energy metabolism, while also playing a pivotal role in neuronal histone acetylation. By directly binding chromatin, ACSS2 serves as a vital link connecting acetic acid metabolism to the regulation of neuronal genes, which is of paramount importance in hippocampal memory consolidation (Mews et al., 2017).

A previous study demonstrated decreased ACSS2 expression in neurons during the acute phase of SAH. Elevated ACSS2 levels have been shown to enhance ATG5-induced autophagy and inhibit apoptosis as a neuroprotective mechanism in the context of SAH (He et al., 2022). In this study, proteomic analysis revealed significant differences in protein expression between SAH mice and their normal counterparts. This is the first study to show that decreased ACSS2 expression in the brains of SAH mice persists beyond the acute phase, indicating a long-term effect. Given the crucial role of ACSS2 in synaptic plasticity and memory, it is plausible that the enduring cognitive impairment observed in SAH mice may be partially attributable to reduced ACSS2 expression levels. Histone acetylation, particularly at telomere-proximal chromatin, is crucial for maintaining telomere stability and preventing DNA damage. Dysfunctional acetylation pathways may exacerbate telomere shortening and genomic instability, creating a feedback loop that further impairs cellular recovery mechanisms (Jezek and Green, 2019). Elevating ACSS2 levels or utilizing acetate supplementation therapy can restore synaptic plasticity and cognitive function in an ACSS2-dependent manner (Lin et al., 2023), offering a possible therapeutic strategy for improving SAH prognosis.

Based on these findings, it is evident that SAH not only presents immediate risks but also leads to long-term consequences that resemble brain aging. It is conceivable that telomere shortening, triggered by excessive oxidative stress during the acute phase of SAH, accelerates brain aging, subsequently affecting normal energy metabolism and signal transduction within the central nervous system. The long-term effects of SAH resemble accelerated brain aging. Metabolic anomalies could be rectified to ameliorate cognitive impairment in the chronic stage of SAH, similar to approaches used to address age-related cognitive decline. Therefore, future studies should explore the potential efficacy of targeting specific metabolic dysfunctions in the aging brain to improve SAH prognosis.

This study has several limitations. Firstly, bleeding volume in SAH mice was not assessed immediately after surgery, because our focus was on long-term SAH prognosis. Secondly, imaging evaluation was not feasible due to experimental limitations. Additionally, the potential influence of hydrocephalus, which may contribute to cognitive decline in animal models of SAH, was not addressed in this study. These limitations should be considered in future studies to further enhance our understanding of the this topic.

## Conclusion

In conclusion, the present study provides evidence that SAH mice display enduring neurocognitive impairment over an extended period of time. Notably, during the chronic stage of SAH, telomere shortening in the brain is closely associated with neurological dysfunction, serving as a predictive marker for unfavorable long-term SAH prognosis. These findings offer promising avenues for the intervention in both the acute and chronic stages of SAH. Moving forward, it is imperative for future SAH studies to not only focus on early brain injury or cerebrovascular spasm, but also to emphasize the significance of the chronic stage, to improve long-term outcomes.

## Data Availability

*The datasets generated and/or analysed during the current study are available in the iProX repository (integrated Proteome resources), project ID: IPX0008486000*.
